# Delayed administration of suramin attenuates peritoneal fibrosis in rats

**DOI:** 10.1186/s12882-019-1597-2

**Published:** 2019-11-14

**Authors:** Chongxiang Xiong, Na Liu, Xiaofei Shao, Sairah Sharif, Hequn Zou, Shougang Zhuang

**Affiliations:** 1 Department of Nephrology, The Third Affiliated Hospital of Southern Medical University, Guangzhou, 510630 China; 2 Department of Medicine, Rhode Island Hospital and Alpert Medical School, Brown University, Providence, RI 02903 USA; 3 Deparment of Nephrology, Shanghai East Hospital, Tongji University School of Medicine, China Shanghai, 200120 China

**Keywords:** Suramin, Peritoneal fibrosis, Epidermal growth factor receptor, Signal transducers, activator of transcription 3, Extracellular signal-regulated kinases ½

## Abstract

**Background:**

Peritoneal fibrosis is the most common complication of peritoneal dialysis, but there is currently no effective treatment. We previously reported that suramin pretreatment prevents the development of peritoneal fibrosis in a rat model of peritoneal fibrosis induced by chlorhexidine gluconate (CG). Here, we further examined the effectiveness of delayed administration of suramin on peritoneal fibrosis and the mechanism (s) involved in this process.

**Methods:**

In the rat model of peritoneal fibrosis induced by CG, suramin or saline was administered at day 21 and 28. All rats were then sacrificed to collect peritoneal tissues for Western blot analysis and histological staining at day 35.

**Results:**

Our results demonstrated that delayed administration of suramin starting at 21 days following CG injection can ameliorate peritoneal damage, with greater efficacy after two injections. Suramin also reduced the expression of α-smooth muscle actin, Collagen 1, and Fibronectin and suppressed phosphorylation of Smad-3, epidermal growth factor receptor (EGFR), signal transducers, activator of transcription 3 (STAT3) as well as extracellular signal-regulated kinases 1/2 (ERK 1/2) in the peritoneum injured with CG. Moreover, delayed administration of suramin inhibited overproduction of transforming growth factor-β1(TGF-β1) and expression of several pro-inflammatory cytokines, including monocyte chemoattractant protein-1, tumor necrosis factor-α, interleukin-1, and interleukin-6.

**Conclusions:**

Our results indicated that suramin can attenuate progression of peritoneal fibrosis by a mechanism involving inhibition of the TGF-β1/Smad3 and EGFR signaling pathways as well as suppression of multiple proinflammatory cytokines. Thus, suramin may have the potential to offer an effective treatment for peritoneal fibrosis.

## Background

Peritoneal dialysis (PD) has been an established technique for renal replacement therapy in end stage of renal disease since the 1970s. However, prolonged exposure to peritoneal dialysis fluid (PDF) results in the alteration of peritoneal morphology and physiology. Over time, this leads to histological changes such as detachment of the mesothelial layer, increased sub-mesothelial extracellular matrix deposition, fibrosis, and angiogenesis [1]. Other factors also contribute to peritoneal membrane structure changes including: peritonitis and the use of bio-incompatible peritoneal dialysis solutions (glucose, glucose degradation products, and advanced glycation end products) [2]. These changes ultimately lead to impairments in solute removal and ultrafiltration failure [3]. Currently there is no effective therapy to prevent peritoneal fibrosis in PD or treat established peritoneal fibrosis.

Suramin has been used in the treatment of African trypanosomiasis and onchocerciasis since 1961 [4, 5]. Emerging evidence has demonstrated suramin’s remarkable anti-neoplastic properties via anti-angiogenic and anti-proliferative pathways. These effects stem from the inhibition of apoptosis, suppression of nuclear factor κ light polypeptide gene enhancer in B cells (NF-κB) activation, reduction of interlerkin-6 (IL-6) and other pro-inflammatory cytokines [6]. In addition, suramin has been used as a synergistic therapy for bladder cancer [7] and a treatment for postoperative glaucoma scarring [8]. Recent studies in animal models have also shown that suramin was effective in the prevention of renal fibrosis [9], suppression of liver and muscle fibrosis [10, 11] and increased control of myocardial inflammation [12]. Mechanistically, suramin attenuates renal fibrosis by inactivation of platelet-derived growth factor receptor (PDGFR), epidermal growth factor receptor (EGFR) and suppression of transforming growth factor-β1(TGF-β1) production [9]; suramin decreases inflammatory responses by suppressing the expression of monocyte chemoattractant protein-1 (MCP-1), regulated on activation upon normal T cell expressed and secreted (RANTES), and intercellular adhesion molecule 1 (ICAM-1) [13]. In cortical neuronal cultures, administration of suramin has also been reported to reduce infarct volume, enhanced anti-angiogenesis and synaptogenesis [14].

Peritoneal fibrosis is a consequence of two main processes: mesothelial cell transformation and peritoneal inflammation. They are triggered by uremia, peritoneal dialysis catheter use, as well as glucose and its degradation products. Injury to mesothelial cells leads to increased production of TGF-β1 and vascular endothelial growth factor (VEGF). As a result, the mesothelial cells are transformed into myofibroblasts, leading to accumulation of extracellular matrix (ECM) [6] and capillary permeability and density are increased, resulting in eventual ultrafiltration failure [6, 15]. In addition, injury to the mesothelial cells causes peritoneal inflammation, which is characterized by macrophage infiltration and overproduction of various cytokines/chemokines [6]. Excessive production of cytokines/chemokines leads to the acceleration of peritoneal fibrosis.

Among the cytokines and growth factors involved in peritoneal fibrosis, TGF-β1 plays a pivotal role in peritoneal fibrosis. It can induce activation of fibroblasts and ECM deposition through activation of several intracellular signaling pathways, including Smad-3 [16], signal transducer and activator of transcription 3 (STAT3) [17], and extracellular signal regulating kinase 1/2 (ERK1/2) [18]. Activation of EGFR can also induce phosphorylation of ERK1/2 and STAT3 [19–21]. As such, EGFR may be involved in the activation of the TGF-β1 signaling pathway and development of tissue fibrosis. In this context, our previous studies have shown that EGFR inhibition suppresses expression of TGF-β1, as well as phosphorylation of Smad3, STAT3, and ERK1/2, and reduces expression of α-smooth muscle actin (α-SMA), collagen type I and fibronectin in injured kidney and cultured renal interstitial fibroblasts [13]. Moreover, pharmacological inhibition of EGFR attenuates development and progression of peritoneal fibrosis in animal models of peritoneal fibrosis induced by chlorhexidine gluconate (CG) or peritoneal dialysates with high glucose [22].

We recently showed that suramin ameliorates peritoneal fibrosis induced by chlorhexidine gluconate (CG) in a rat model when given before peritoneal damage [23]. Here we further explore the therapeutic effects of suramin on peritoneal fibrosis in the same model by delayed administration of suramin starting at day 21 after injection with CG.

## Methods

### Antibodies and chemicals

Antibodies for p-STAT3, STAT3, p-Smad-3, Smad-3, p-ERK1/2, ERK1/2, p-EGFR were purchased from Cell Signaling Technology (Danvers, MA). Antibodies for fibronectin, collagen 1(A2), GAPDH, and EGFR were purchased from Santa Cruz Biotechnology, Inc. (Santa Cruz, CA). Suramin, antibodies for α-SMA and all other chemicals were purchased from Sigma-Aldrich (St.Louis, MO).

### Animal model

All the experiments were performed in Thirty-six 6–8 week old Male Sprague-Dawley rats (170 ± 10 g) purchased from B&K laboratory animal Corp (Shanghai, China). Rats were randomly separated into six groups with 6 rats in each group: Sham (control), Sham with 20 mg/kg suramin at day 21, **s**ham with 20 mg/kg suramin at day 21 and 28, CG, CG with 20 mg/kg suramin at day 21, and CG with 20 mg/kg suramin at day 21 and 28. To investigate the effect of suramin on established peritoneal fibrosis, rats were given 1 ml/100 g body weight of 0.1% CG (Sigma, USA) daily by intraperitoneal injection for 21 days to induce peritoneal fibrosis. The rats in the Sham group were administrated with intra-peritoneal injection of saline and suramin for the same time at the same dosages. All rats were sacrificed at day 35 after initial injection of CG by using overdose of sodium pentobarbital at 200 mg/kg and the parietal peritoneum distant from the injection points was harvested for further analyses. All the study were performed according to the procedures approved by the animal ethic committee of Tongji University (Shanghai, China).

### Histology and Immuno-histochemical staining

Four-micrometer-thick samples of peritoneal tissue were stained with hematoxylin and eosin and Masson’s trichrome to determine the pathological changes and peritoneum thickness. Immuno-histochemical staining was performed as previously described [23, 24].

### Elisa

TGF-β1, MCP-1, IL-1β, TNF-α, IL-6 protein expression levels in peritoneal tissue were analyzed using the Colorimetric Sandwich ELISA Kits (R&D System, Minneapolis, MN). Cytokine detection was performed in accordance with the manufacturer’s instructions.

### Immunoblot analysis

Protein expression from peritoneal tissue lysates were analyzed by Western blot analysis according to our previous studies [24]. The blots were quantitated by using NIH Image J software (National Institutes of Health).

### Statistical analysis

All the experiments (Immunoblot analysis and ELISA) were conducted at least three times to ensure definitive results and accuracy. Data are depicted as the means ± S.E.M. for each group. Intergroup comparisons were performed by one-way ANOVA. Multiple means were compared using Tukey’s test. Comparison between two groups was made by Student’s T-tests. Statistically significant differences between mean values were marked in each graph. *P* < 0.05 was considered significant.

## Results

### Suramin treatment ameliorates CG-induced pathological changes in the peritoneal membrane and downregulates the expression of α-SMA, collagen 1, and fibronectin in a time-dependent manner

**Fig. 1:**
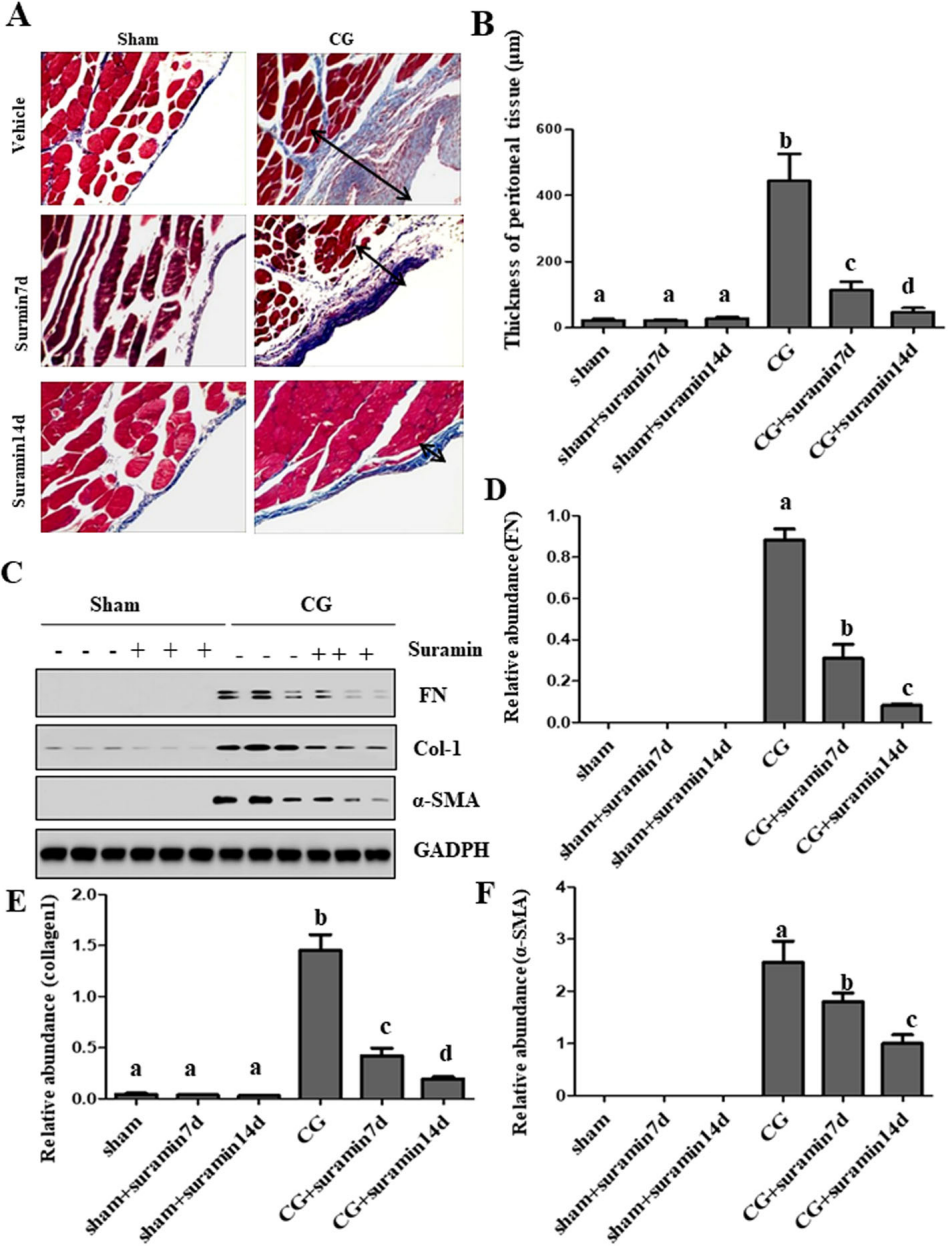
Suramin treatment ameliorates CG-induced pathological changes in the peritoneal membrane and downregulates the expression of α-SMA, collagen 1, and fibronectin in a time-dependent manner. Photomicrographs illustrating Masson trichrome staining of peritoneum in the rat model of CG-induced peritoneal fibrosis administered with suramin at different times. Masson trichrome-positive areas decreased after 2 injection of suramin (**a**). The thickness of compact area (average value) measured from ten random fields in different group (**b**). Peritoneal lysates were subjected to immunoblot analysis with antibodies for fibronectin, collagen1, α-SMA, and GAPDH (**c**). Expression levels of fibronectin (**d**), collagen1 (**e**), α-SMA (**f**) were quantified by densitometry and normalized with GAPDH. Data are represented as mean ± S.E.M (*n* = 6). Means with different lowercase letters are significantly different from one another (*P* < 0.05)

To determine the effect of suramin on CG-induced peritoneal fibrosis (PF), we established a rat model of PF by daily intraperitoneal injection of CG to the animals for 3 weeks and then treated it with suramin on day 21 and 28, respectively. Then, we performed a histological assessment of the peritoneum by Masson trichrome staining. As shown in Fig. 1a&b, Masson staining demonstrated that the thickness of the submesothelial compact zone increased while greater collagen deposition was seen in the peritoneal tissue of the CG group when compared with the sham group. Masson trichrome-positive areas were markedly decreased after day 28 of treatment, whereas, peritoneal thickness remarkably decreased in the suramin treatment group (Fig. 1b). The pathological changes induced by CG include increased thickness of submesothelial compact areas and expression of α-SMA, collagen 1, and fibronectin [25]. We used immunoblot analysis to examine the impact of suramin treatment on the expression of those extracellular matrix components. In accordance with the histological changes, suramin treatment also significantly decreased the expression of fibronectin (Fig. 1c, d) and α-SMA (Fig. 1c, f). The expression of collagen1 (Fig. 1c, e) also decreased in a time-dependent manner after suramin treatment.

### Suramin inhibits the activation of the TGF-β1-smad3 signaling pathway

**Fig. 2:**
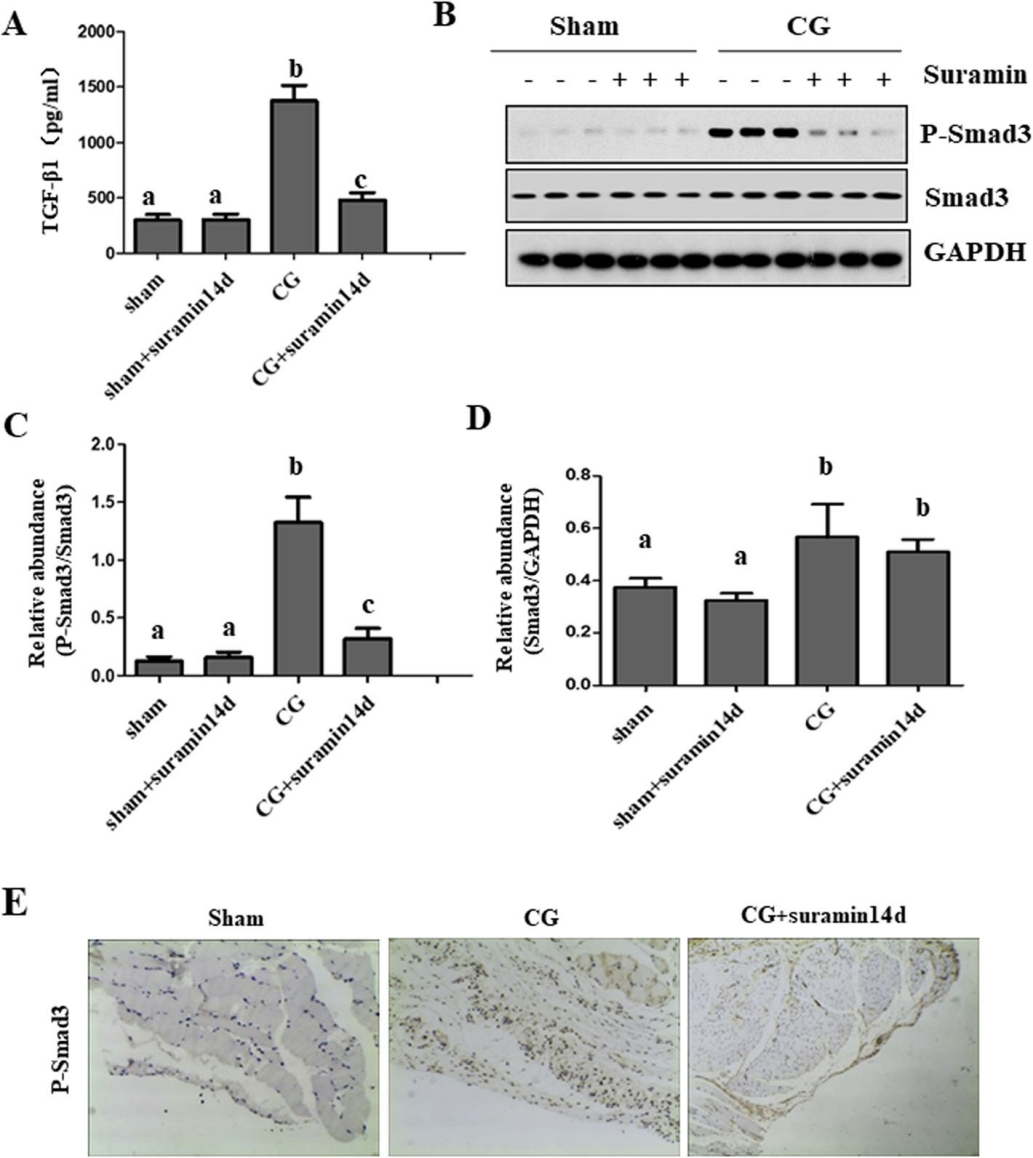
Suramin inhibits the expression of TGF-β1 and Smad3 phosphorylation in a rat model of peritoneal fibrosis. TGF-β1 levels in lysates from peritoneal tissues were determined by the ELISA (**a**). Peritoneal lysates were subject to immunoblot analysis with antibodies to phosphorylated Smad3 (p-Smad), Smad3, or GAPDH (**b**). Expression levels of p-Smad3 were quantified by densitometry and normalized with total Smad3 (**c**). Expression levels of Smad3 were quantified by densitometry and normalized with GAPDH (**d**). Photomicrographs illustrating immunochemistry staining of p-Smad-3 in the peritoneum (**e**). Data are represented as the mean ± S.E.M. (*n* = 6). Means with different lowercase letters are significantly different from one another (*P* < 0.05)

Activation of the TGF-β1/Smad3 pathway is one of the major mechanisms involved in the pathogenesis of PF [26, 27]. To examine whether suramin treatment affects the activation of the TGF-β1/Smad3 signaling pathway, we first measured the levels of TGF-β1 through the ELISA. In addition, we examined the expression of phosphorylated-Smad3 (p-Smad3) using immunoblot analysis and immunochemistry staining. Our findings demonstrated that expression of TGF-β and p-Smad3 dramatically increased following CG intra-peritoneal injection. Suramin treatment significantly suppressed their expression (Fig. 2a-c). Notably, total Smad-3 levels also slightly increased in the peritoneum of CG-treated animals, but suramin treatment did not affect its expression (Fig. 2b, d). Immunohistochemical staining also displayed marked positive expression of p-Smad3 in the peritoneum of the PF rat model, and suramin treatment partially reduced its expression (Fig. 2e). These results suggest that suramin is effective in attenuating PF through the inhibition of TGF-β1 expression and Smad3 activation.

### Suramin treatment suppresses the phosphorylation of EGFR and inhibits the expression of p-Stat3 and p-ERK1/2 in peritoneal tissue

**Fig. 3:**
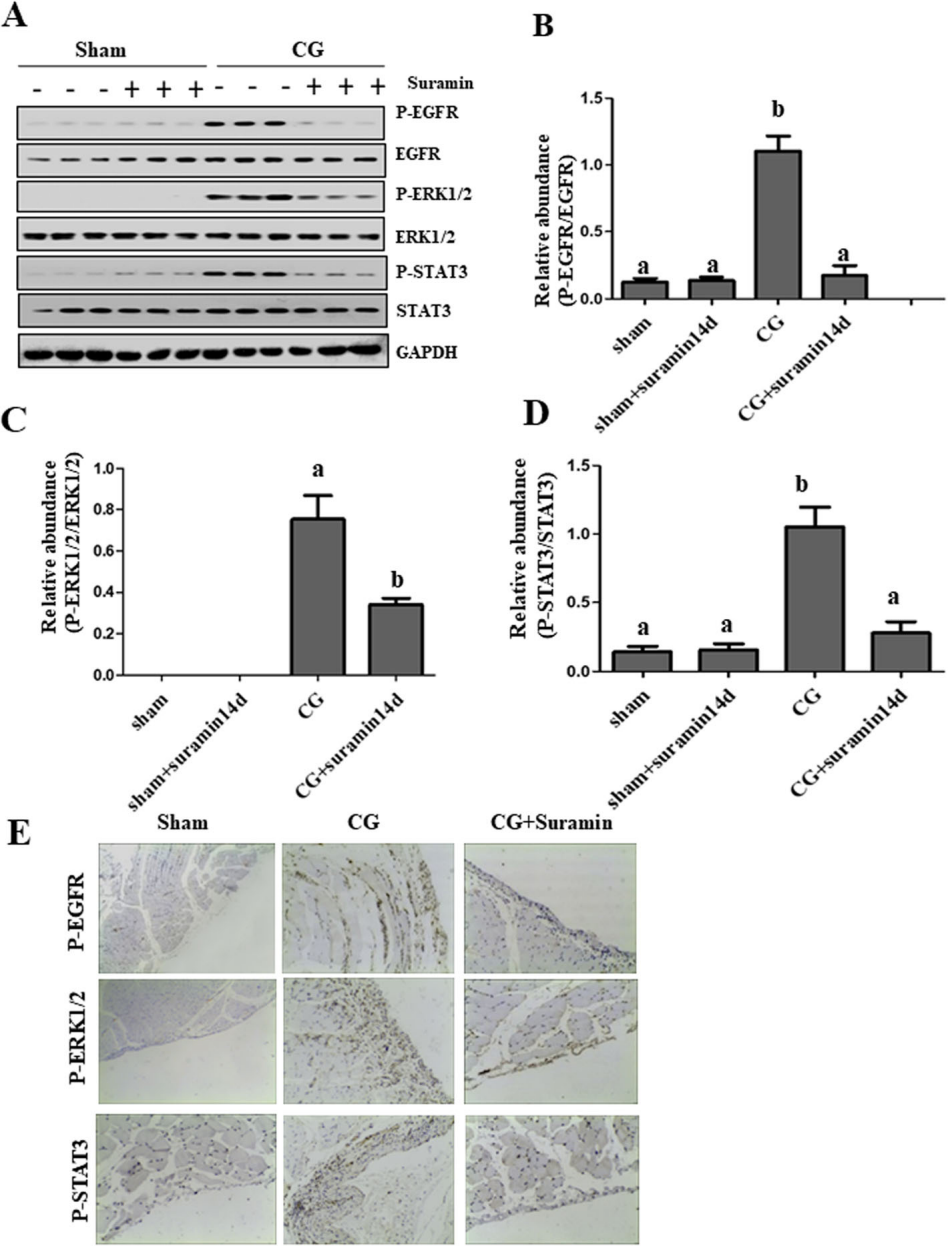
Suramin treatment suppresses the phosphorylation of EGFR, Stat3 and ERK1/2 in peritoneal tissue. Peritoneal lysates were subjected to immunoblot analysis with antibodies to phosphorylated EGFR (p-EGFR), phospho-ERK1/2 (p-ERK1/2), phosphorylated Stat3 (p-STAT3), EGFR, ERK1/2, Stat3, or GAPDH (**a**). Expression levels of p-EGFR were quantified by densitometry and normalized with total EGFR (**b**). Expression levels of p-ERK1/2 were quantified by densitometry and normalized with total ERK1/2 (**c**). Expression levels of p-Stat3 were quantified by densitometry and normalized with total Stat3 (**d**). Data are represented as the mean ± S.E.M. (*n* = 6). Means with different lowercase letters are significantly different from one another (*P* <0.05). (**e**) Representative photomicrograph of immunochemistry staining of p-EGFR, p-ERK1/2, p-Stat3 in the submesothelial compact zone

Increasing evidence has shown that EGFR plays an important role in renal fibrogenesis [20]. To elucidate the role of P-EGFR in peritoneal fibrosis, we tested the expression of p-EGFR by immunoblot analysis and immunohistochemical staining. As shown in Fig. 3a, e, expression of p-EGFR was markedly increased in peritoneal tissue injured by CG, whereas, treatment with suramin reduced p-EGFR expression despite CG exposure (Fig. 3, a and b). These results indicate that activation of EGFR may be involved in the development of PF following CG injection. Furthermore, suramin may reduce peritoneal fibrosis through a mechanism involved in the suppression of EGFR activation.

Pathologic activation of Stat3 and ERK1/2 by phosphorylation (p-Stat3 and p-ERK1/2) occurs in organ fibrosis, including renal fibrosis [28]. To determine the role of p-Stat3 and p-ERK1/2 in PF, we examined the expression of these two molecules using immunoblot analysis and immunohistochemical staining. Expression levels of P-Stat3 and P-ERK1/2 significantly increased in the CG group and downregulated following suramin administration (Fig. 3, a, c, d). Immunohistochemistry staining further showed that P-Stat3 and p-ERK1/2 were mainly expressed in the submesothelial compact areas. Only weak or undetectable positive staining of these two molecules was observed in the sham group and sham + suramin group (Fig. 3e). This data suggests that suramin treatment may decrease PF via suppression of Stat3 and ERK1/2 signaling pathways.

### Suramin treatment inhibits the expression of pro-inflammatory cytokines in rats with peritoneal fibrosis

**Fig. 4:**
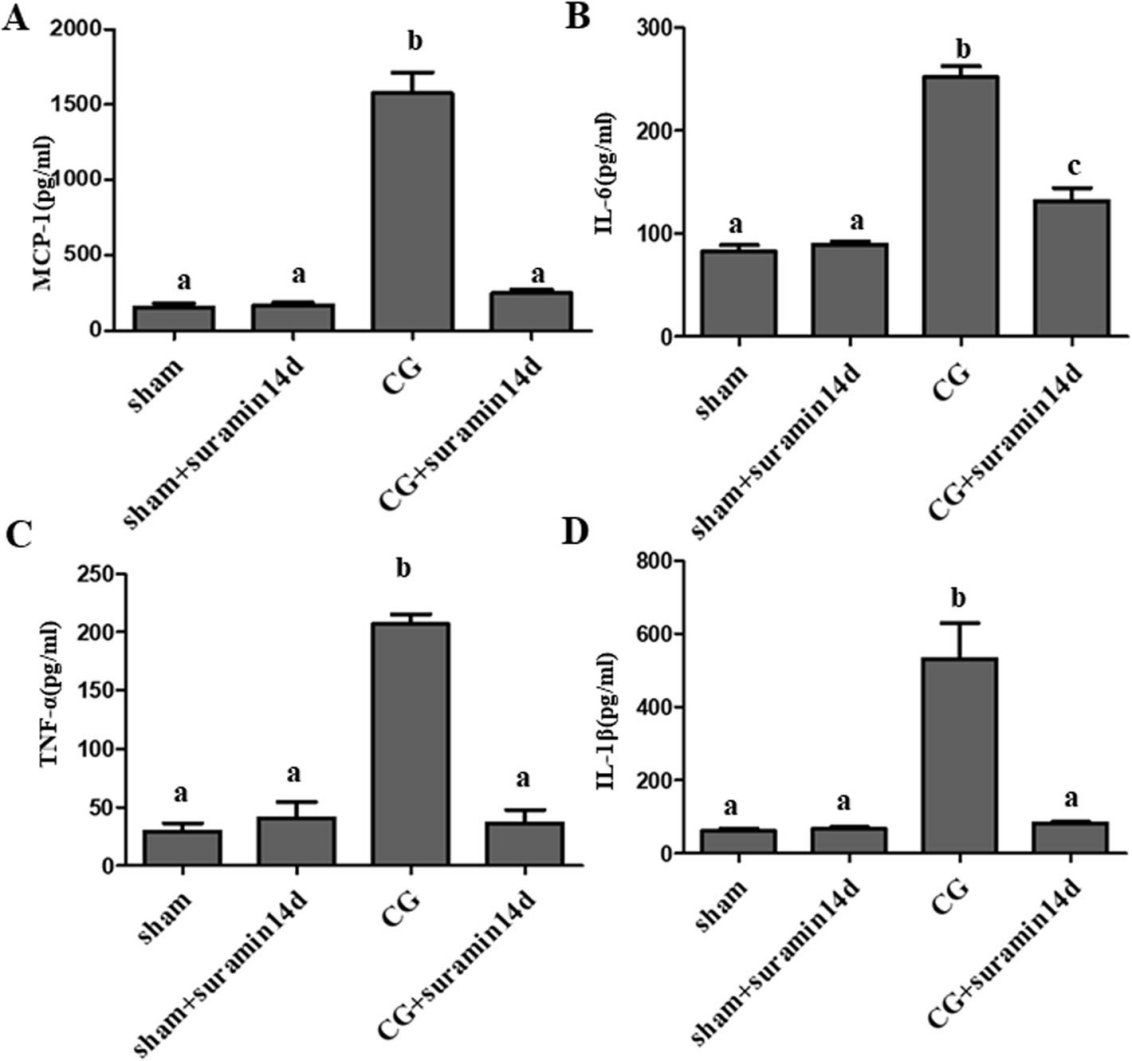
Suramin suppresses the expression of MCP-1, TNF-α, IL-1β, and IL-6 in a rat model of CG-induced peritoneal fibrosis. Peritoneal lysates were subjected to ELISA as described under Materials and Methods. The expression levels of MCP-1 (**a**), IL-1β (**b**), TNF-α (**c**), and IL-6 (**d**) are indicated and compared to the control. Data is represented as the mean 6 S.E.M. (*n* = 6). Means with different lowercase letters are significantly different from one another (*P*<0.05)

Pro-inflammatory cytokines are associated with the progression of PF. We examined the effect of suramin treatment on pro-inflammation cytokines using the ELISA. Treatment with suramin resulted in reduction in pro-inflammatory cytokines like MCP-1, IL-6, TNF-α and IL-1β (Fig. 4, a-d) over time in the rat model of PF induced by CG. Thus, suramin administration was effective in decreasing the expression of pro-inflammatory cytokines. These results demonstrate that suramin has the potential to alleviate PF by inhibiting the production of pro-inflammatory cytokines.

## Discussion

While PD is an effective form of renal replacement therapy, long-term exposure of the peritoneal membrane to PDF is associated with permanent membrane damage. The peritoneal membrane is composed of a single layer of mesothelial cells resting on an underlying interstitial substrate consisting of collagen fibrils and peritoneal capillaries. Multiple factors affect peritoneal membrane health and lead to damage in PD patients over time. These factors include: uremia, PD catheter use, and PDF [29]. Prolonged exposure of the peritoneum to PDF ultimately leads to peritoneal remodeling, ultrafiltration decline, and eventual technique failure. Some constituents of PDF such as buffer, low pH, glucose concentration, and glucose degradation products are harmful to the peritoneum and contribute to peritoneal fibrosis [30]. Despite advances in understanding its causes, the exact mechanism leading to PF remains unclear, and no effective therapies are currently available for its treatment. In this study, we demonstrated that delayed administration of suramin ameliorates PF induced by CG in a rat model, suggesting that suramin may be an agent with therapeutic potential in the treatment of peritoneal fibrosis.

Suramin is a polysulfonated naphthylurea that inhibit multiple growth factors and cytokines by binding to their receptors. Given that Suramin can block the stimulatory effect of TGF-β1 on muscle-derived fibroblasts in vitro [31], we examined the effect of suramin on the expression of TGF-β1 and activation of Smad3 in the injured peritonium. Our results showed that the expression of TGF-β1 and p-Smad3 were reduced in the peritoneum with suramin treatment, accompanied by decreased expression of α-SMA, collagen1 and fibronectin, three components of the ECM. These results are consistent with our previous observations that suramin is able to block TGF-β1 expression and Smad3 phosphorylation, leading to the attenuation of renal fibrosis and PF [23]. As TGF-β1/Smad signaling is the most important cascade in EMT [6] and Smad3 activation promotes PF [32], our results suggests that suramin may suppress the synthesis of ECM and reduce PF development by targeting TGF-β1/Smad signaling.

Suramin may also suppress peritoneal fibrosis through a Smad-independent mechanism. Previous studies have shown that TGF-β1 can induce activation of STAT3 and ERK1/2 signaling pathways and that STAT3 and ERK1/2 have a synergistic effect on proliferation of cardiac fibroblasts and production of collagens [33]. In the current study, we demonstrated that suramin inhibition of TGF-1 signaling is accompanied by dephosphorylation of STAT3 and ERK1/2 in the injured peritoneum. This suggests that reduced phosphorylation of STAT3 and ERK1/2 secondary to inhibition of TGF-β1 signaling by suramin may serve as a mechanism by which suramin attenuates peritoneal fibrosis [34]. However, we can’t exclude the possibility that suramin also inhibits these two pathways by suppressing other cellular membrane growth/cytokine receptors, in particular, EGFR. In support of this hypothesis, our data showed that suramin inhibits CG-induced EGFR phosphorylation as well as STAT3 and ERK1/2 phosphorylation. Moreover, our previous studies demonstrated that pharmaceutical and genetic blockade of EGFR leads to dephosphorylation of STAT3 and ERK1/2 and attenuation of renal fibrosis [13]; pharmaceutical inhibition of EGFR/ERK signaling is associated with reduction of peritoneal fibrosis [22]. Taken together, these findings suggest that suramin may inhibit Smad3-independent profibrotic signaling pathways by targeting multiple membrane receptors, including TGF-β1 receptor and EGFR.

It is well known that pro-inflammatory cytokines play a critical role in the pathogenesis of PF. Long-term PD results in continuous inflammation of peritoneal tissue due to the exposure to a high concentration of glucose and its degradation products. In addition, there is an increase in the production of cytokines including TNF-α, IL-1β, MCP-1, IL-6 in the peritoneum after injury [34–36]. Proinflammatory cytokines can induce the epithelial to mesenchymal transition (EMT), a cellular process with the loss of cell-to-cell and cell-to-matrix interactions [37]. The transformed mesothelial cells participate in inflammation, angiogenesis, and produce ECM leading to fibrosis formation. Our findings demonstrated that suramin treatment reduces the expression of the aforementioned pro-inflammatory cytokines, suggesting another potential mechanism by which suramin attenuates peritoneal fibrosis.

## Conclusions

Taken together, we demonstrated that administration of suramin can ameliorate PF in a rat model induced by CG injection. The effect of suramin on established PF is in part due to reduction in the expression of both the TGF-β1 and inhibition of P-smad3 signaling pathways. Additionally, suramin treatment may also affect Smad-independent mechanisms associated with peritoneal fibrosis such as inhibition of Stat3 and ERK1/2 phosphorylation. We therefore conclude that suramin may represent a potential novel drug therapy for delaying or inhibiting the progression of PF in patients subject to long-term PD therapy. It has a further advantage of having already been approved for use in humans in the treatment of helminth infection and certain forms of cancer, suggesting that human trials might be safely undertaken after appropriate review by institutional review boards.

## Data Availability

The datasets created during and/or analysed during the current study will be available from the corresponding author on reasonable request. There are no security, licensing, or ethical issues related to these data.

## Abbreviations

CG: Chlorhexidine gluconate
ECM: Extracellular matrix
EGFR: Epidermal growth factor receptor
EMT: Epithelial to mesenchymal transition
ERK1/2: Extracellular signal regulating kinase 1/2
ESRD: End stage of renal disease
ICAM-1: Intercellular adhesion molecule-1
IL: Interleukin
MCP-1: monocyte chemoattractant protein-1
NF-kB: Nuclear factor κ light polypeptide gene enhancer in B cells
PD: Peritoneal dialysis
PDF: Peritoneal dialysis fluid
PDGFR: Platelet-derived growth factor receptor
RANTES: Regulated on activation upon normal T cell expressed and secreted
RRT: Renal replacement therapy
STAT3: Signal transducer and activator of transcription 3
TGF-β1: Transforming growth factor-β1
TNF-α: Tumor necrosis factor-α
VEGF: Vascular endothelial growth factor
α-SMA: α-smooth muscle actin
